# Antimicrobial Effect of Common Bacterial Pigments on Clinically Significant Microorganisms

**DOI:** 10.1155/sci5/3951925

**Published:** 2025-07-01

**Authors:** Afroz Salman, Biranthabail Dhanashree, Himani Kotian

**Affiliations:** ^1^Department of Microbiology, Kasturba Medical College Mangalore, Manipal Academy of Higher Education, Manipal, India; ^2^Department of Community Medicine, Kasturba Medical College Mangalore, Manipal Academy of Higher Education, Manipal, India

**Keywords:** antimicrobial effect, minimum inhibitory concentration, prodigiosin, pyocyanin, violacein

## Abstract

The aim of this research is to investigate the antimicrobial characteristics of bacterial pigments. Pigments are vibrant metabolites generated by bacteria, offering defence against radiation, sunlight, stress and competing microorganisms. Bacterial pigments have various applications in cosmetics, pharmaceuticals and food sectors due to their antimicrobial, anticancer and antioxidant properties. In this cross-sectional in vitro study, we used 30 isolates each of *Enterococcus faecalis*, *Klebsiella* spp. and *Candida* spp. isolated from human urine, blood and pus samples to study the antimicrobial effect of pigments produced by *Serratia marcescens* (prodigiosin), *Chromobacterium violaceum* (violacein) and *Pseudomonas aeruginosa* (pyocyanin). At the suitable pH and temperature, pigment-producing bacteria were mass-cultured in nutrient broth. Pigments were extracted from *S. marcescens* and *C. violaceum* cultures using acetone and methanol. Pyocyanin from *P. aeruginosa* was extracted by using hydrochloric acid and chloroform. Extracted pigments were dried and characterised by mass spectrometry. The antimicrobial activity of the crude pigments was determined by the agar dilution method using Muller Hinton agar (MHA) plates for bacterial isolates, and Sabouraud's Dextrose agar (SDA) for *Candida* spp. *E. faecalis* ATCC 29212, *K. pneumonia* ATCC 700603 and *C. albicans* ATCC 14053 were used as standards. The minimum concentration of the pigment that supressed the growth of microorganism on the MHA/SDA agar plate was considered as minimum inhibitory concentration (MIC). Data were analysed using the Chi-square statistical method. *E. faecalis*, which were resistant to teicoplanin, vancomycin, norfloxacin and penicillin, were found susceptible to pyocyanin (*p* < 0.05). Multidrug-resistant *Klebsiella* spp. were susceptible to pyocyanin, violacein and prodigiosin (*p* < 0.05). *Candida* spp. resistant to caspofungin, micafungin, showed susceptibility to prodigiosin, and those isolates resistant to amphotericin B showed susceptibility to pyocyanin (*p* < 0.05). Among all clinical isolates, *Klebsiella* spp. from urine exhibited the highest resistance to the pigments studied. Among the pigments studied, violacein and prodigiosin exhibited superior antimicrobial and antifungal properties. Therefore, violacein and prodigiosin could serve as an alternative antimicrobial substance for addressing multidrug-resistant pathogenic microorganisms.

## 1. Introduction

Any material that absorbs light at specific wavelength to change the colour of reflected or transmitted light is called a pigment. Production of pigment may be extracellular or intracellular. Diffusible pigments are water-soluble and non-diffusible pigments are water-insoluble [1]. Chromogenic bacteria are microorganisms that form coloured colonies. The pigments produced by these bacteria safeguard them from radiation, oxidation stress, extremes of temperatures and drying. Bacterial pigment synthesis and demonstration of its potential for various applications is an emerging field of research. They are known to have antibacterial, antiviral, antioxidant and anticancer properties in addition to being colouring compounds utilised in the food and cosmetics industries [2].

Research work on ‘natural bacterial pigments' are doubled in the last few years, because use of synthetic dyes in industries ends up by dumping toxic chemical waste into water sources leading to blooming of algae, ultimately increasing greenhouse gas emissions [3]. Pigments extracted from plants do have antimicrobial property. But plant pigments get denatured by pH change and fail to provide batch reproducibility. So, researchers have shown interest in microbial pigments [3]. Because of their short life cycle, minimal sensitivity to seasonal and climatic fluctuations and ease of production, bacteria provide unique advantages over other types of microorganisms. These physical traits make bacterial pigments seem like a promising area for new biotechnological uses, like creation of functional foods and innovative medications for biomedical treatments [4]. Natural pigments obtained from plants and animals are non-toxic, non-carcinogenic and biodegradable and thought to be safe. According to reports, bacterial pigments such as prodigiosin, pyocyanin, violacein and indigo dyes are effective medicinal agents [5, 6] as these act as immunosuppressive agents during organ transplantation by inhibiting T cell proliferation.

The red bioactive pigment known as prodigiosin has the strongest antibacterial action, followed by orange, yellow and green pigments. These bioactive compounds will provide promising future in biomedical research. However, when monitoring the antimicrobial activities of microbial pigments, researchers use non-pure pigments that can give variable results [7]. A new marine strain of *Salinicoccus* spp. that produces pinkish-orange pigment was identified by Srilekha et al. They found that the pigment had the strong antibacterial action against *Staphylococcus aureus* and weak activity against *Klebsiella pneumonia* and *Pseudomonas aeruginosa* [8]. A few studies have also tested the antibacterial effect of pigments from diverse group of marine bacteria on standard strains of Gram-positive and Gram-negative bacteria but not on clinical isolates [4, 8, 9]. However, pigments produced by *Micrococcus luteus* and *P. aeruginosa* have been tested for their antimicrobial activities against very few human pathogens isolated from wound and urinary tract infections [4].

Prodigiosin is known to have antibacterial and antiparasitic effects. However, the exact mechanism of its action is still not known. Prodigiosin has been reported as a promising broad-spectrum antimicrobial agent. [10]. Violacein and pyocyanin pigments also have antibacterial, antiviral and antibiofilm activities [6]. Prodigiosin was shown to be one of the most successful adjuvants for overcoming colistin resistance in multidrug-resistant (MDR) *Klebsiella* and *Acinetobacter* species [11].

Prodigiosin, as a chaotropic stressor, damages the bacterial or fungal plasma membrane, causing the loss of vital elements including proteins and ions and obstructs metabolism [12, 13]. Although *Salmonella* Typhimurium was more resistant to the pigment, when compared to *E.coli*, prodigiosin is known to stop the growth of biofilms and virulence factors in both bacteria [14]. According to the earlier findings, the isolated prodigiosin demonstrated high antimicrobial activity against *Salmonella* Typhimurium, *Listeria monocytogenes, Bacillus cereus, P. aeruginosa*, *S. aureus* and *Vibrio parahaemolyticus*. Additionally, the prodigiosin's minimum inhibitory concentrations (MICs) ranged from 3 μg/mL to 30 mg/mL [15].

Majority of the previous studies have investigated the effect of bacterial pigments on standard strains of bacteria, fungi or a handful of clinical isolates. In this era of increasing multidrug resistance and availability of very few safe antibacterial agents for the treatment of MDR pathogens, it is necessary to have alternative therapeutic agents. Since clinical strains of bacteria frequently exhibit antibiotic resistance and there are currently limited novel medications to treat these infections, we intend to explore the antimicrobial effect of bacterial pigments on clinically significant *E. faecalis*, *Klebsiella* spp. and *Candida* spp.

## 2. Materials and Methods

### 2.1. Study Setting, Design and Duration

This observational cross-sectional study of 8-month duration was carried out between August 2023 and March 2024 at the Department of Microbiology. The Institutional Ethical Committee (IEC) provided ethical permission for this research [IEC KMC MLR 05/2023/232].

### 2.2. Microbial Isolates Used in the Study

A total of 30 isolates each of *E. faecalis*, *Klebsiella* spp. and *Candida* spp. obtained from clinical samples were included in the study by following convenient non-random sampling method. Reference strains of *E. faecalis* ATCC 29212*, K. pneumonia* ATCC 700603*, Candida albicans* ATCC 14053, *P. aeruginosa* ATCC 27853, *Serratia marcescens* MTCC 97T and *Chromobacterium violaceum* MTCC 2656 were also used in the study. All the chemicals, media, antibiotic discs and ATCC reference strains for the study was procured from Hi-Media Laboratories Pvt Ltd. Mumbai, India, unless otherwise specified. *Serratia marcescens* MTCC 97T and *Chromobacterium violaceum* MTCC 2656 were procured from Microbial Type Culture Collection and Gene Bank, Chandīgarh, India. All bacterial and fungal isolates were identified by biochemical reaction, and antibiotic susceptibility was done by using VITEK-2 system (bioMérieux, USA). Pure cultures of these bacteria were preserved in 20% glycerol broth at −80°C [16], for further workup.

### 2.3. Extraction of Pigment From Chromogenic Bacteria

In order to determine the best suited pH and temperature needed for pigment synthesis, chromogenic bacteria were cultivated in nutrient broth with varying pH values of 7, 8 and 9, incubated for 48 h at two distinct temperatures, 25°C and 37°C. Amount of growth was detected by measuring absorbance at 600 nm by a spectrophotometer [17]. The most suitable temperature for *S. marcescens* and *C. violaceum* was 27°C and 37°C, respectively, and the pH was 7. Pyocyanin was best produced by *P. aeruginosa* at 37°C and pH 8. These conditions were adapted for large-scale pigment production.

Colonies of *S. marcescens* and *C. violaceum* from nutrient agar were inoculated into 150 mL of freshly prepared nutrient broth with pH 7 and incubated for 48 h at 27°C and 37°C, respectively. Broth culture of *S. marcescens* and *C. violaceum* cultures were centrifuged for 20 min at 7500 rpm, the supernatant was disposed of and cell pellet was collected. One millilitre of a 3:1 acetone and methanol mix was added to the cell pellets, subjected to a freeze–thaw cycle and vortexed to ensure homogeneity. The mixture was centrifuged at 7000 rpm for 15 min to separate the colourless cell debris from the coloured solvent extract. Coloured supernatant was filtered through Whatman No. 1 filter paper, transferred to a sterile glass Petri plate and dried overnight in an incubator at 37°C. Dried pigments were scraped, and their dry weight was measured using a weighing balance and expressed as mg/150 mL, later dissolved in methanol to get a concentration of 1 mg/mL [17]. Dried pigments were stored at 4°C until further use.

Colonies of *P. aeruginosa* were inoculated into 150 mL of nutrient broth with pH 8 and incubated at a temperature of 37°C for 72 h. Since *P. aeruginosa* produces diffusible pigment, the top layer of the *P. aeruginosa* broth culture was taken after it was centrifuged for 15 min at 1000 rpm. The membrane filter (0.45 μm) was used to filter this supernatant. The filtrate was treated with chloroform in a 1:2 ratio and vortexed for 20 s. A blue chloroform layer appears, which was transferred to another flask, treated with equal volume of 0.2 N HCl and vortexed till a pink colour pigment transfers into upper aqueous phase. It was centrifuged for 5 min at 10000 g. The absorbance of this pigment was measured at 520 nm in a spectrophotometer. The concentration of pyocyanin (μg/mL) pigment was calculated by multiplying the optical density (OD520) with an extinction coefficient of 17.072. This pink pigment in aqueous layer was subsequently treated with 1 N NaOH drop by drop till the colour changed to blue. Two characteristics that set pyocyanin apart are its dual solubility and its apparent blue-to-pink colour shift [18].

### 2.4. Biophysical Characterisation of Microbial Pigments

#### 2.4.1. LC/MS Analysis of Crude Microbial Pigments

To investigate the quality and possible constituents in the crude extract of the pigments, Thermo Fisher's Orbitrap (Q Exactive) mass spectrometry coupled with Vanquish liquid chromatography was used. The crude pigments were analysed by LC-MS at Manipal School of Life Sciences, Manipal, India. Capillary column fused with silica gel (250 × 4.6 mm) was used as the stationary phase, and methanol and chloroform mixture (1:1) was used as the mobile phase. A gradient elution solvent used involved water with 0.1% formic acid (solvent A) and methanol with 0.1% formic acid (solvent B). The gradient starts from a high percentage of aqueous solvent to a high percentage of organic solvent over time to separate pigments based on hydrophobicity. Based on the mass-to-charge (m/z) ratios of protonated or deprotonated molecular ions of the pigments, precursor ions selected for analysis included chorismic acid, phenazine-1-carboxylic acid (PCA) for pyocyanin, L-tryptophan for violacein, 4-methoxy-2,2′-bipyrrole-5-carbaldehyde (MBC) and 2-methyl-3-n-amylpyrrole (MAP) for prodigiosin. Collision energies were adjusted for each pigment to produce characteristic fragment ions in higher-energy collisional dissociation (HCD) or collision-induced dissociation (CID) modes. Typical collision energies range from 20 to 40 eV but are adjusted based on the pigment's stability and fragmentation pattern. In our experiment, the collision energies were as follows: pyocyanin: 30–60 eV, violacein: 25 eV, prodigiosin: < 100 eV. Under the experimental condition, the reproducible retention energies used were pyocyanin: 11.64 min, violacein: 15.6 min, prodigiosin: 10.071 min. The MS data were processed and the LC/MS fragmentation and molecular profiles were compared with commercial spectral libraries/databases containing reference spectra of known bacterial pigments. Thermo Fisher Mass Frontier software version 8.1 was used in predicting the fragment ions, confirming pigment identity and calculation of the molecular weight of the compound. For statistical and bioinformatics analysis, METLIN database was used.

### 2.5. Testing of Antimicrobial Activity of the Pigments

The antimicrobial activity/MIC of pigments was determined by agar dilution method [19]. Muller Hinton agar (MHA) containing different concentrations of pigments (1 to 7 mg/mL) were spot inoculated with 2 µL of clinical isolates and ATCC strains whose turbidity was adjusted to 0.5 McFarland standard. These inoculated plates were incubated at 37°C for 24 h and observed for growth. The development of one or more colonies or a thin film of growth at a specific concentration in the pigment-containing MHA indicated resistance to the pigment concentration. The minimum concentration of the pigment that suppresses the growth of test strain on MHA plates was considered as MIC. To test the antifungal effect of pigment on *Candida* spp*.,* Sabouraud's Dextrose agar (SDA) was used instead of MHA. Reference strains of *E. faecalis* ATCC 29212*, K. pneumonia* ATCC 700603 and *C. albicans* ATCC 14053 were used as standards/positive control in agar dilution technique [19]. Uninoculated MHA and SDA containing various concentrations of pigments were used as sterility control. MHA and SDA plates without pigments, inoculated with standard strains of bacteria/*Candida* spp. were used as growth control. MIC testing for each isolate was repeated three times.

To rule out the inhibitory effects of solvents used in pigment extraction (acetone, methanol, chloroform, 0.2 N HCl, 1 N NaOH), they were incorporated at 10%, 20%, 30%, 40% and 50% concentrations in MHA/SDA plates. Two microlitres of reference strains of *E. faecalis* ATCC 29212*, K. pneumonia* ATCC 700603 and *C. albicans* ATCC 14053 whose turbidity was adjusted to 0.5 McFarland standard were inoculated and plates were incubated at 37°C for 24 h. Inoculated plates were observed for inhibition of growth. These tested concentrations of solvents did not inhibit the growth of reference strains.

### 2.6. Antimicrobial Susceptibility Testing

The susceptibility pattern of clinical isolates of *E. faecalis*, *Klebsiella* spp. and *Candida* spp. was studied by the Kirby Bauer disc diffusion method using the antibiotics and antifungals mentioned in Tables 1, 2 and 3, respectively. Reference strains of *E. faecalis* ATCC 29212, *K. pneumonia* ATCC 700603 and *C. albicans* ATCC 14053 were used as controls. Results were read as susceptible, intermediate or resistant using Clinical and Laboratory Standards Institute (CLSI) guidelines [20].

### 2.7. Statistical Analysis

All tests were conducted in triplicate and displayed as mean values; individual tests with a standard deviation of less than 0.5% were deemed significant. For inferential statistical data analysis, Jamovi (version 2.3.28), a free and open statistical platform, was used. The growth inhibition of *E. faecalis*, *Klebsiella* spp. and *Candida* spp. by pigments were compared with antimicrobial resistance patterns of routinely used antimicrobial agents using the Chi-square test (*p* value < 0.05 is considered as statistically significant).

## 3. Results and Discussion

### 3.1. Distribution of Bacterial and Fungal Isolates in Clinical Samples and Their Antimicrobial Susceptibility

**Figure 1:**
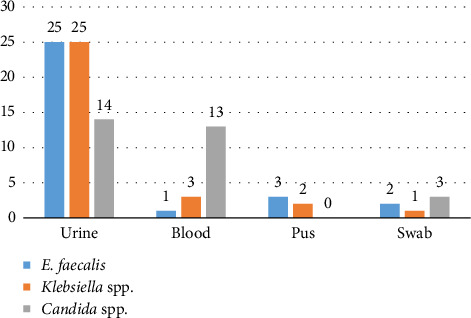
Distribution of bacterial and fungal isolates in clinical samples.

In this study, a total of 90 clinical isolates were collected from different clinical samples such as urine, wound swab, blood and pus. Among the 30 isolates of *E. faecalis,* 25 (83%) were from urine, 3 (10%) were from pus, 2 (7%) from wound swab and 1 (3%) was from blood. Of 30 isolates of *Klebsiella* spp., 24 (81%) were from urine, 2 (6%) from pus, 1 (3%) from wound swab and 3 (10%) were from blood. Out of the 30 isolates of *Candida* spp., 14 (43%) were from urine, 3 (17%) from wound swab and 13 (40%) were from blood (Figure 1).

Antimicrobial susceptibility patterns to commonly used antibiotics and antifungals mentioned in Tables 1, 2 and 3 were studied by disc diffusion method. Of the 30 isolates of *E. faecalis,* 50% were resistant to levofloxacin, 46.6% to ciprofloxacin, 2% to vancomycin and 3.3% to daptomycin and teicoplanin. The current scenario of drug susceptibility for *E. faecalis* is similar to earlier findings [21], whereas maximum resistance to vancomycin was reported from Europe and resistance was found less in geographical areas having more rainfall [21, 22]. Thus, our findings show that resistance pattern of microorganism varies with the geographical area and antibiotic usage. Among the 30 isolates of *Klebsiella* spp., 30% were resistant to ofloxacin, 83.3% to ampicillin and 43.3% to ertapenem. The drug-resistant pattern for *Klebsiella* spp. in India [23] was found to be comparable to our investigation, and similar results were also obtained in other countries, such as Italy [24]. Urine-derived *Klebsiella* spp. exhibited maximum carbapenem resistance (33.33%) among all clinical isolates in our investigation. However, Iraq was estimated to have 40% carbapenem resistance [25]. Similarly, amphotericin B (26.6%) and caspofungin (23.3%) showed the highest levels of resistance among *Candida* species. Indian research findings were comparable to ours [26]. Menoufia University study conducted internationally revealed a resistance pattern that was also comparable to ours [27]. The overall pattern of antibiotic resistance in our isolates and those found in previous research points to a slow increase in antibiotic resistance, necessitating the use of a more potent new antimicrobial agent for therapy.

### 3.2. Modulation of Conditions for Growth and Pigment Production

The growth and pigment production were improved by adjusting the pH of culture medium and temperature used for growing the pigment-producing bacteria. *S. marcescens* exhibited the maximum growth and best pigment production at pH 7 and 27°C. Similar findings were reported by Bhagwath et al. [28]. *C. violaceum* demonstrated maximum growth and pigment production at 37°C and pH 7, which was similar to work done at the University of Technology, Malaysia [29]. In *P. aeruginosa*, maximum growth and pigment production were obtained at 37°C and pH 8, as reported by earlier workers also [30].

### 3.3. Mass Spectrometry (LC/MS)

**Figure 2:**
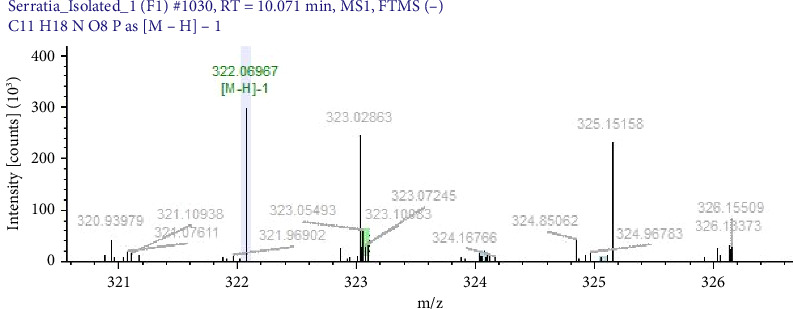
Spectrograph of prodigiosin as detected by LC-MS. Peak marked in green colour representing the molecular weight (323.10) of prodigiosin.

**Figure 3:**
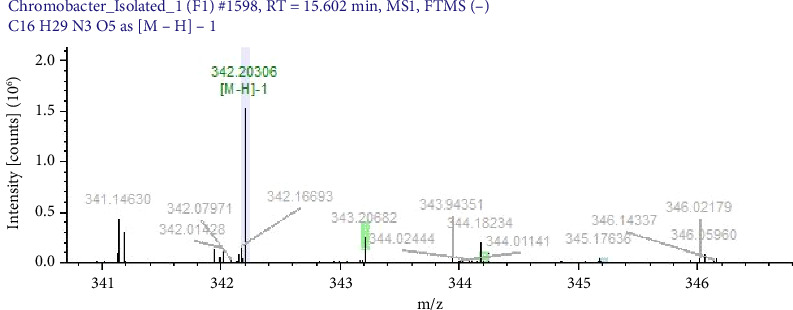
Spectrograph of violacein as detected by LC-MS. Peak marked in green colour representing the molecular weight (343.20) of violacein.

**Figure 4:**
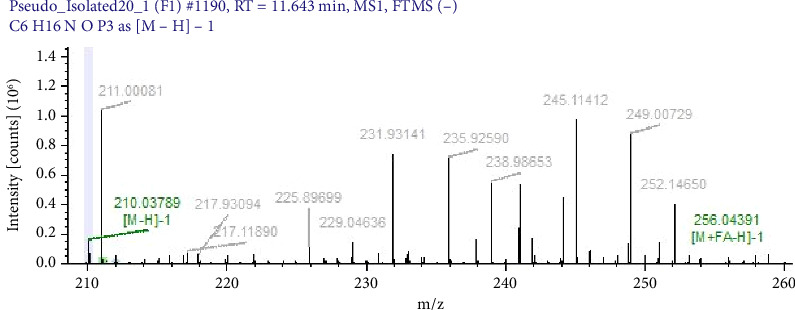
Spectrograph of pyocyanin as detected by LC-MS. Peak marked in green colour representing the molecular weight (210.03) of pyocyanin.

Pigments produced by standard strains were characterised by mass spectrometric methods to confirm possible compounds and molecular structure. The antimicrobial compounds in bacterial pigments were confirmed as pyocyanin, violacein and prodigiosin by LC/MS. Further, they were analysed by mass/charge ratio (m/z), and their molecular weight were also calculated. Pigment extracted from *S. marcescens* was confirmed as prodigiosin (mol.wt 323 g/mol), pigment from *C. violaceum* as violacein (mol.wt 343.34 g/mol) and pigment of *P. aeruginosa* as pyocyanin (mol.wt 210 g/mol). Earlier studies by Paul et al. 2024 [31], Yusof et al. 2012 [29] and Devnath et al. [18] showed similar findings. The molecular weights of pigments extracted and used in MIC studies are shown in Figures 2, 3 and 4.

### 3.4. Antimicrobial Activity of Bacterial Pigments on Clinical Isolates

The MICs of prodigiosin, violacein and pyocyanin were determined for clinical bacterial isolates (*n* = 60), *Candida* spp. (*n* = 30) along with the reference strains using the agar dilution method. This experiment was repeated three times, and results were reproducible. Figures S1to S3 illustrate how various pigment concentrations inhibited clinical bacterial strains, *Candida* spp and reference strains as determined by the agar dilution method. Furthermore, the growth of reference strains was not inhibited by solvents employed in pigment extraction, even at 50% concentration. These findings clearly demonstrate that solvent residues had no effect on the MIC results. MICs of prodigiosin, pyocyanin and violacein for standard strain of *E. faecalis* ATCC 29212 and *C. albicans* ATCC 14053 were 2.5, 2.0 and 2.5 mg/mL, respectively. MICs of prodigiosin, pyocyanin, and violacein for standard strain of *K. pneumoniae* ATCC 700603 were 3.0, 4.0 and 3.0 mg/mL, respectively.

Violacein and prodigiosin demonstrated the highest antimicrobial activity against clinical strains of *E. faecalis* at MIC 2.5 mg/mL, while pyocyanin displayed the lowest activity at MIC 5 mg/mL (Table 4). Of the 30 isolates of *E. faecalis,* 29(96.6%) isolates had MIC of 2.5 mg/mL for prodigiosin, 28 (93.3%) had MIC of 2 mg/mL for violacein and 27 (93.3%) showed MIC of 3 mg/mL for pyocyanin (Table 4). *E. faecalis* (*n* = 7), which showed resistance to all three pigments, were from urine samples. Most of the studies done previously have used only standard strains of Gram-positive and Gram-negative bacteria to study the antimicrobial effect of prodigiosin [14]. However, in addition to the reference strains, we evaluated 30 isolates of *E. faecalis*, *Klebsiella* spp. and *Candida* spp. to provide a more comprehensive understanding of the inhibitory effects of bacterial pigments.

Among 30 isolates of *Klebsiella* spp., 22 (73.3%) had MIC of 3.5 mg/mL for prodigiosin, 19 (63.3%) had MIC of 3.5 mg/mL for violacein and 23(76.6%) had MIC of 5 mg/mL for pyocyanin (Table 4). *Klebsiella* spp., which showed resistance to all the three pigments, were from urine (*n* = 18), blood (*n* = 2) and pus (*n* = 3) samples. As carbapenem resistance in *Klebsiella* spp. is increasing [32], prodigiosin may be considered as a promising antibacterial agent for carbapenem-resistant *Klebsiella*.

Of the 30 clinical isolates of C*andida* spp., 26 (86.6%) had MIC of 2.5 mg/mL for prodigiosin, 25 (83.3%) had MIC of 2.5 mg/mL for violacein and 26 (86.6%) had MIC of 5 mg/mL for pyocyanin (Table 4). The *Candida* spp., which was resistant to all three pigments, were from urine (*n* = 10), swab (*n* = 2) and pus (*n* = 3) samples. Antifungals like caspofungin and micafungin are known for its toxicity [33]. Findings of our study show that prodigiosin can be considered as an alternative antifungal agent as it is being used as a safe food colouring agent [2]. Similar results to ours have been found in earlier research by Tejaswini et al. from India [34] and the University of Chittagong in Bangladesh [30]. An earlier study by Ji et al. (2019) reports prodigiosin's MIC range from 3 µg/mL to 30 mg/mL [15]. But they have studied the antimicrobial effect of pigments on standard strains and not the clinical strains.

Research shows that prodigiosin has a wide range of antibacterial properties. But the precise way it works is still unclear. As a chaotropic stressor, prodigiosin damages the bacterial or fungal plasma membrane, causing the loss of vital elements including proteins and ions and obstructs metabolism [12, 13]. Although *Salmonella* Typhimurium was more resistant to the pigment, when compared to *E. coli*, prodigiosin is known to stop the growth of biofilms and virulence factors in both bacteria [14].

To the best of our knowledge, melanin has been well investigated for its antimicrobial properties, but pyocyanin pigment has not. Pyocyanin (5 mg/mL) applied topically as an ointment has been proven in prior research to effectively eradicate *S. aureus*, *K. pneumoniae* and *C. albicans* from rabbits' infected wounds [35]. Pyocyanin's antibacterial properties were proposed to be mediated by either disrupting the microorganisms' redox state or mitochondrial respiratory chain or cell wall/membranes.

Violacein's antibacterial activity is thought to involve mechanisms like disruption of cell membranes inducing oxidative stress and inhibiting nucleic acid and protein synthesis. Violacein has been reported to have an inhibitory effect on *Candida albicans* and *Aspergillus niger* with MICs ranging from 4.375 mg/mL and 8.75 mg/mL [36]. The MIC data for violacein was reported to range from 5.7 μg/mL to 20.0 μg/mL for different reference strains of bacteria [37].

Thus, from the results of our study and that of previous studies, we conclude that prodigiosin from *S. marcescens* has a good inhibitory effect on the clinical isolates from urine samples, whereas violacein from *C. violaceum* and pyocyanin from *P. aeruginosa* have good inhibitory effect on clinical isolates from blood and pus samples. However, more research is needed to determine the precise process by which these pigments interact with microorganisms and cause their destruction.

### 3.5. Comparison of Inhibitory Activity of Pigment and Resistance Patterns of Common Antimicrobials Tested

The antimicrobial activities of the pigment on *E. faecalis*, *Klebsiella* spp. and *Candida* spp. were compared with antimicrobial resistance patterns of routinely used antimicrobial agents. *E. faecalis*, which were resistant to teicoplanin, vancomycin, norfloxacin and penicillin, were found susceptible to pyocyanin (Table 1). *Klebsiella* spp., which were resistant to cefixime & ertapenem, were susceptible to pyocyanin, those resistant to ciprofloxacin were susceptible to violacein and those isolates resistant to meropenem, amikacin and nalidixic acid were susceptible to prodigiosin. These results were statistically significant (*p* < 0.05), as shown in Table 2. Similarly, *Candida* spp. resistant to caspofungin, micafungin, showed susceptibility to prodigiosin, and those isolates resistant to amphotericin B showed susceptibility to pyocyanin, as shown in Table 3. Our results show that violacein (5 mg/mL) and prodigiosin (4.5 mg/mL) were bactericidal for a higher percentage of clinical isolates at lower concentrations than pyocyanin (7 mg/mL), as shown in Table 4. Earlier study from India has shown antibacterial effect of pigments extracted from soil bacteria on clinical strains (one each) of *Escherichia coli*, *Salmonella* Typhi, *S. aureus*, *Pseudomonas* spp. and *Candida* spp. [38].

The majority of previous research has examined how bacterial pigments affect reference bacterial strains, fungus or a small number of clinical isolates. These studies have used disc diffusion or well diffusion method and have not compared the MIC results of pigments with resistance to commonly used antibiotics for therapy [15, 35–38]. However, in our study, we have used 90 clinical and three reference strains of microorganisms to investigate the effects of bacterial pigments, and compared the MIC of pigment obtained by agar dilution method with the resistance pattern of routinely used antimicrobials. Furthermore, pyocyanin has not been investigated as an antibacterial agent, but melanin pigment has been investigated for its antimicrobial action in previous studies [18, 35]. We have chosen the pigments of *S. marcescens, C. violaceum* and *P. aeruginosa* since these bacteria that produce pigments also infect immunocompromised humans. Thus, our findings pave the way for additional research on pigment's efficacy, either alone as a potential drug or in conjunction with other commonly used antimicrobials.

Thus, in this era of increasing drug resistance in clinical isolates, bacterial pigments need to be considered as an alternative for treatment of infectious diseases. Since bacterial pigments are already being used as food colouring agent, its side effects are minimal [30]. These pigments with nanoparticle caps may be used to create antimicrobial agents that combat pathogenic strains resistant to antibiotics. The use of pigment as antibiotics is limited by a number of factors, including its large-scale manufacturing, selective narrow spectrum of activity, gradual degradation that may produce toxic chemicals, limited bioavailability and need for delivery mechanisms to reach the target organs or cells. [39]. As a result, our study suggests carrying out comprehensive animal studies and human clinical trials to demonstrate the safety and efficacy of pigments as a substitute antibiotic for treating infections.

## 4. Limitations

Clinical strains of *Candida* spp., *Klebsiella* spp. and *E. fecalis* were investigated to determine the antibacterial activity of crude pigment extract. To rule out any limiting activity against specific bacteria, a greater number of distinct Gram-positive, Gram-negative and fungal isolates must be evaluated using pure extracts due to the limited spectrum of action of these pigments. Therefore, there is room for more research in a multicentric study that uses additional isolates from diverse clinical samples and refined pigment extracts. Clinical studies on humans and animals are necessary to determine the biological potential of these pigments and to shed information on the possible therapeutic uses of both new and established bacterial pigments.

## 5. Conclusions

Three bacterial pigments, namely, prodigiosin produced by *S. marcescens,* violacein by *C. violaceum* and pyocyanin by *P. aeruginosa*, were mass extracted. Antimicrobial effects of these pigments were studied on clinical isolates of *E. faecalis*, *Klebsiella* spp. and *Candida* spp. It was found that all three pigments showed antimicrobial effect. Pyocyanin was found to be effective against vancomycin, teicoplanin, norfloxacin and penicillin-resistant *E. faecalis* and carbapenem-resistant *Klebsiella* spp. (*p* < 0.05). Prodigiosin was highly effective against carbapenem and aminoglycoside-resistant *Klebsiella* spp. (*p* < 0.05). Violacein very effectively inhibited the growth of fluoroquinolones-resistant *Klebsiella* spp. Prodigiosin showed effective antifungal activity against caspofungin and micafungin-resistant *Candida* spp. Pyocyanin had effective antifungal action against amphotericin B resistant *Candida* isolates. Therefore, once its bioavailability and adverse effects have been examined and validated by animal research as well as clinical trials, bacterial pigment may be utilised as a substitute for antimicrobials to inhibit the growth of microorganisms resistant to multiple drugs.

## Figures and Tables

**Table 1:** Comparison of antibiotic resistance with pigment susceptibility in *E. faecalis*.

Antibiotics tested	*E. faecalis*					
	Prodigiosin		Pyocyanin		Violacein	
	*x* ^2^	*p*	*x* ^2^	*p*	*x* ^2^	*p*
Benzyl penicillin	1.88	0.3	4.2	0.117	2.8	0.2
Ampicillin	0.3	0.8	4.2	0.117	1.43	0.4
HLG	1.6	0.2	1.6	0.2	0.03	0.84
Ciprofloxacin	2.2	0.3	3.7	0.15	0.46	0.7
Levofloxacin	1.6	0.4	5.0	0.08	2.14	0.34
Erythromycin	0.3	0.8	2.4	0.29	3.21	0.20
Linezolid	0.5	0.7	0.5	0.7	0.5	0.7
Daptomycin	0.2	0.9	1.4	0.4	3.7	0.15
Teicoplanin	0.1	0.9	14.5	**0.001**	0.153	0.92
Vancomycin	0.2	0.8	14.5	**0.001**	0.23	0.88
Tetracycline	2.4	0.2	1.8	0.3	1.01	0.60
Tigecycline	2.1	0.3	3.2	0.2	2.14	0.34
Nitrofurantoin	0.7	0.6	0.7	0.6	2.61	0.27
Norfloxacin	0.9	0.6	5.8	**0.05**	0.77	0.6
Penicillin	0.7	0.6	1.4	**0.001**	0.76	0.6

*Note:* A statistically significant *p* value < 0.05 was observed. Bold values represent statistically significant *p* values (less than or equal to 0.05).

**Table 2:** Comparison of antibiotic resistance with pigment susceptibility *Klebsiella* spp.

Antibiotics tested	*Klebsiella* spp.					
	Prodigiosin		Pyocyanin		Violacein	
	*x* ^2^	*p*	*x* ^2^	*p*	*x* ^2^	*p*
Ampicillin	1.4	0.4	0.6	0.7	2.1	0.3
Amoxicillin/clavulanic acid	1.2	0.5	0.7	0.6	1.0	0.5
Piperacillin/tazobactam	0.6	0.4	0.14	0.7	0.1	0.6
Cefuroxime	1.5	0.4	0.09	0.9	1.4	0.4
Cefuroxime/axetil	1.5	0.4	0.09	0.9	1.4	0.4
Ceftriaxone	0.6	0.4	0.66	0.4	0.1	0.6
Cefoperazone/sulbactam	1.15	0.5	0.24	0.88	1.1	0.5
Cefepime	3.0	0.2	0.21	0.8	1.6	0.4
Cefixime	1.7	0.4	1.9	**0.03**	1.3	0.5
Ertapenem	2.8	0.2	1.9	**0.03**	1.0	0.5
Imipenem	1.6	0.2	0.03	0.8	0	0.9
Meropenem	2.4	**0.03**	2.0	0.3	0.1	0.9
Amikacin	1.9	**0.03**	0.24	0.88	0.7	0.7
Gentamycin	4.4	0.1	2.0	0.3	0.8	0.6
Ciprofloxacin	2.3	0.12	0.2	0.6	0.8	**0.03**
Tigecycline	0.9	0.6	4.16	0.12	1	0.6
Trimethoprim/sulfamethoxazole	0.8	0.6	0.3	0.82	0.2	0.5
Nalidixic acid	4.9	**0.01**	1.5	0.4	1.1	0.5
Norfloxacin	0.9	0.6	0.8	0.6	1.2	0.2
Ofloxacin	2.3	0.3	0.2	0.8	2.6	0.4

*Note:* A statistically significant *p* value < 0.05 was observed. Bold values represent statistically significant *p* values (less than or equal to 0.05).

**Table 3:** Comparison of antifungal resistance with pigment susceptibility in *Candida* spp.

Antibiotics tested	*Candida* spp.					
	Prodigiosin		Pyocyanin		Violacein	
	*x* ^2^	*p*	*x* ^2^	*p*	*x* ^2^	*p*
Fluconazole	2.6	0.2	1.2	0.5	1.1	0.5
Voriconazole	3.6	0.1	1.1	0.5	4.0	0.13
Caspofungin	5.7	**0.05**	1.8	0.3	0.7	0.6
Micafungin	5.4	**0.05**	0.4	0.8	0.9	0.6
Amphotericin B	2.3	0.3	2.2	**0.03**	0.7	0.6
Flucytosine	2.6	0.2	1.2	0.5	0.6	0.7

*Note:* A statistically significant *p* value < 0.05 was observed. Bold values represent statistically significant *p* values (less than or equal to 0.05.

**Table 4:** Comparison of antimicrobial activity of prodigiosin, pyocyanin and violacein among clinical isolates.

Isolates tested (*n* = 90)	MIC of prodigiosin mg/mL							MIC of pyocyanin mg/mL							MIC of violacein mg/mL						
	1	2	2.5	3	3.5	4	4.5	1	2	3	4	5	6	7	1	2	2.5	3	3.5	4	5
*E. faecalis* (*n* = 30)	0	0	29	30	30	30	30	0	0	27	28	30	30	30	0	28	28	30	30	30	30
*Klebsiella* spp. (*n* = 30)	0	0	0	0	22	30	30	0	0	0	0	23	23	30	0	0	0	0	22	30	30
*Candida* spp. (*n* = 30)	0	0	26	26	26	26	30	0	0	0	0	26	26	26	0	0	25	25	30	30	30

## Data Availability

The data that support the findings of this study are available from the corresponding author upon reasonable request.

## Supporting Information

Figures S1, S2 and S3 are available as supporting information.

Figure S1: A representative picture showing the effect of different concentrations of pigments on *Klebsiella* spp. as determined by agar dilution method.

Figure S2: A representative picture showing the effect of different concentrations of pigments on Enterococcus faecalis as determined by agar dilution method.

Figure S3: A representative picture showing the effect of different concentrations of pigments on *Candida* spp. as determined by agar dilution method.
