# Supplementary material for: Antimicrobial Effect of Common Bacterial Pigments on Clinically Significant Microorganisms
Source: Scientifica (Cairo). 2025 Jul 1;2025:3951925. doi: 10.1155/sci5/3951925 (PMC12256167; doi:10.1155/sci5/3951925)

**Figure S1**: A representative picture showing effect of different concentration of pigments on *Klebsiella* spp. as determined by agar dilution method

**Control 2mg/ml 7mg/ml**


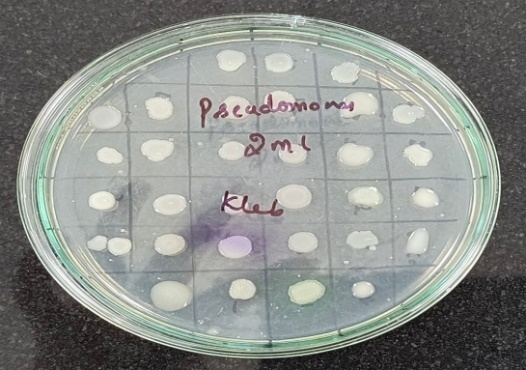

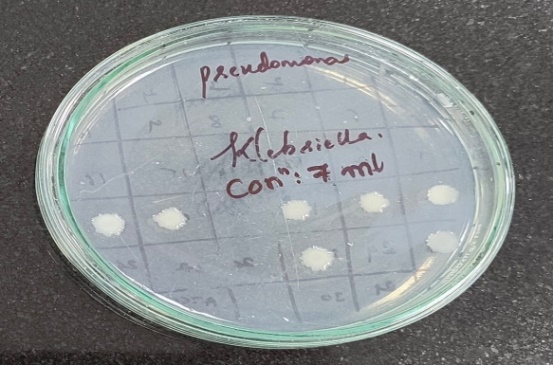

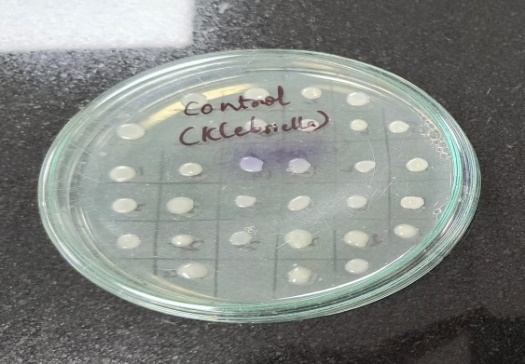


**A**. Increase in pyocyanin concentration inhibits the growth of *Klebsiella* spp*.* and Reference strain


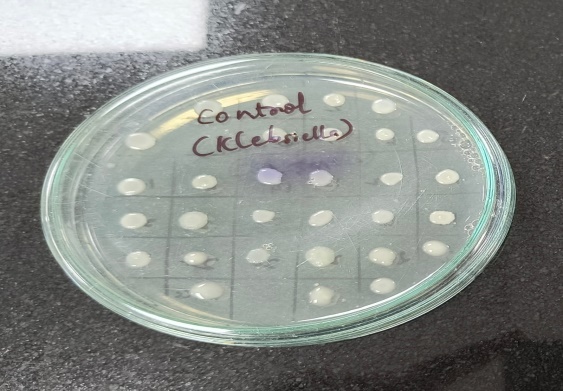


**Control 2mg/ml 4mg/ml**


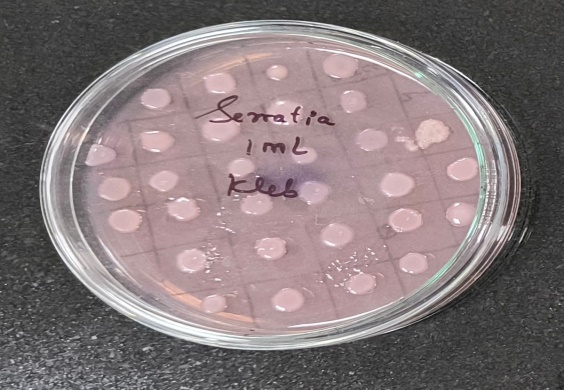

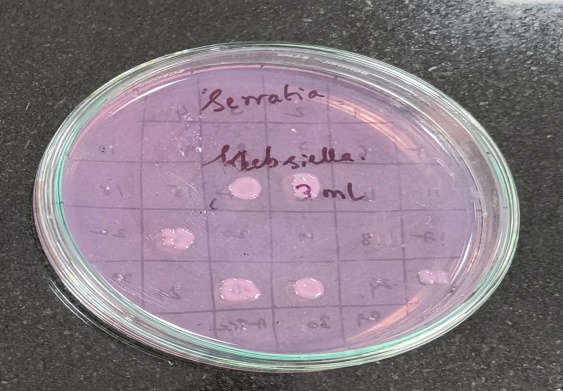


**B**. Increase in prodigiosin concentration inhibits the growth of *Klebsiella* spp. and Reference strain


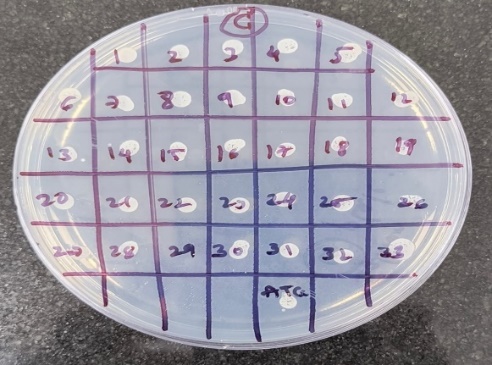

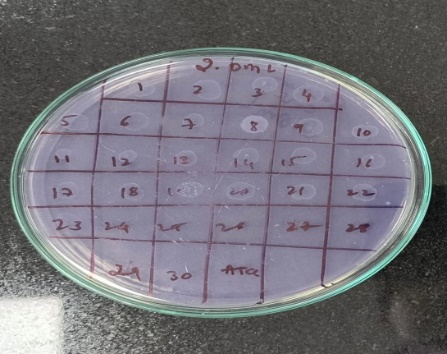

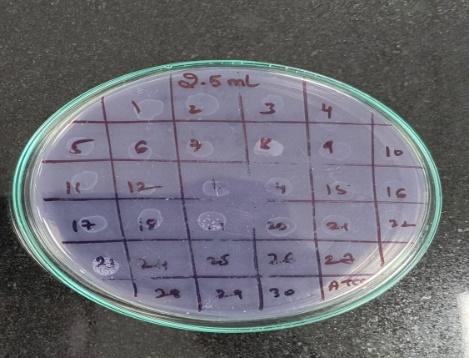


**Control 2mg/ml 4mg/ml**

**C**. Increase in violacein concentration inhibits the growth of *Klebsiella* spp. and Reference strain

**Figure S2**: A representative picture showing effect of different concentration of pigments on *Enterococcus faecalis* as determined by agar dilution method


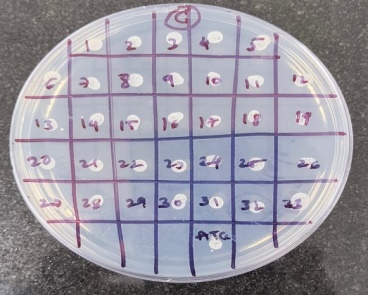

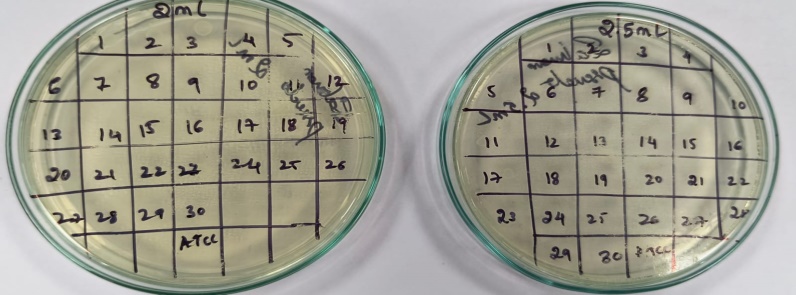

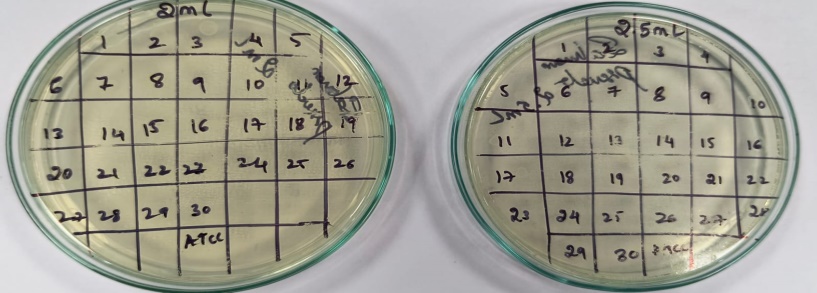


**Control 2mg/ml 5mg/ml**

**A**. Increase in pyocyanin concentration inhibits the growth of *E. faecalis* isolates and Reference strain


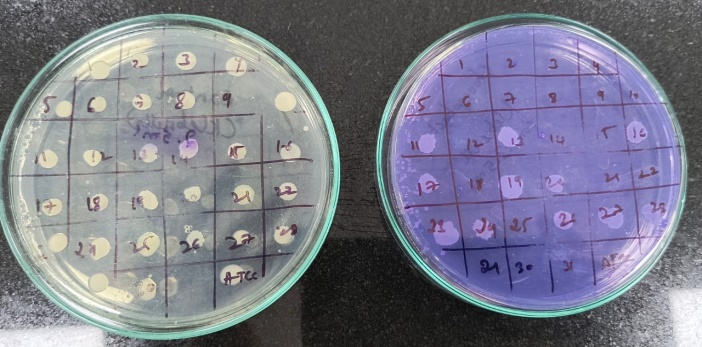

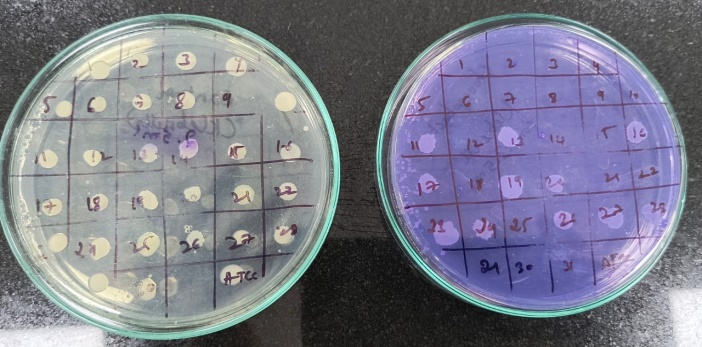


**Control 2mg/ml 2.5mg/ml**


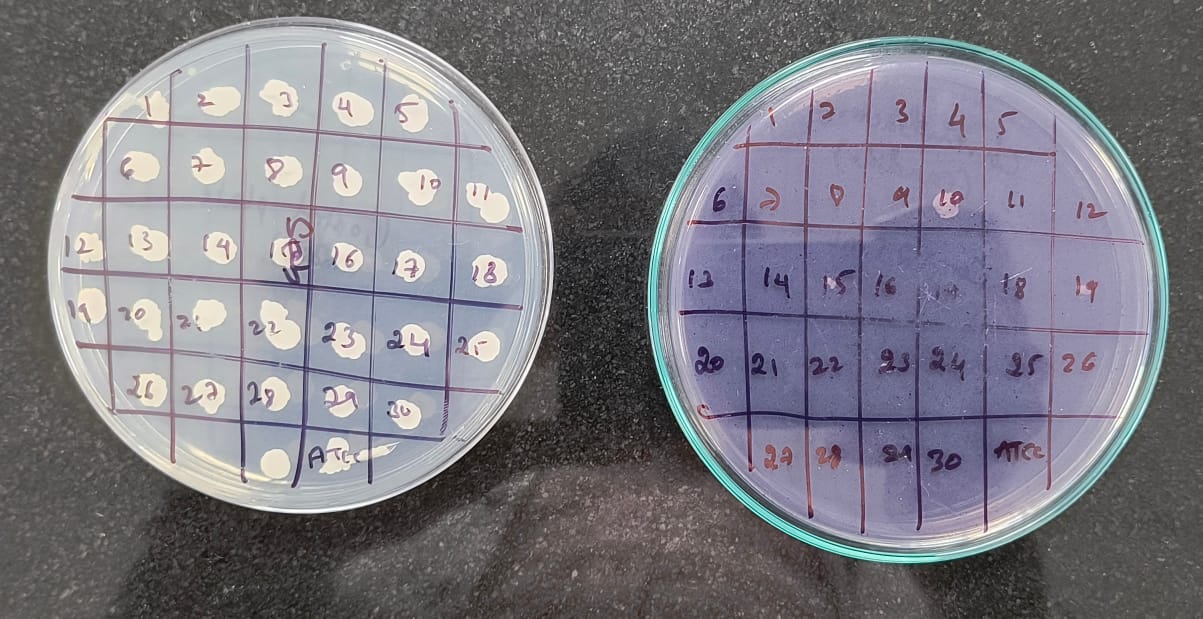


**C**. Increase in violacein concentration inhibits the growth of *E. faecalis* isolates and Reference strain


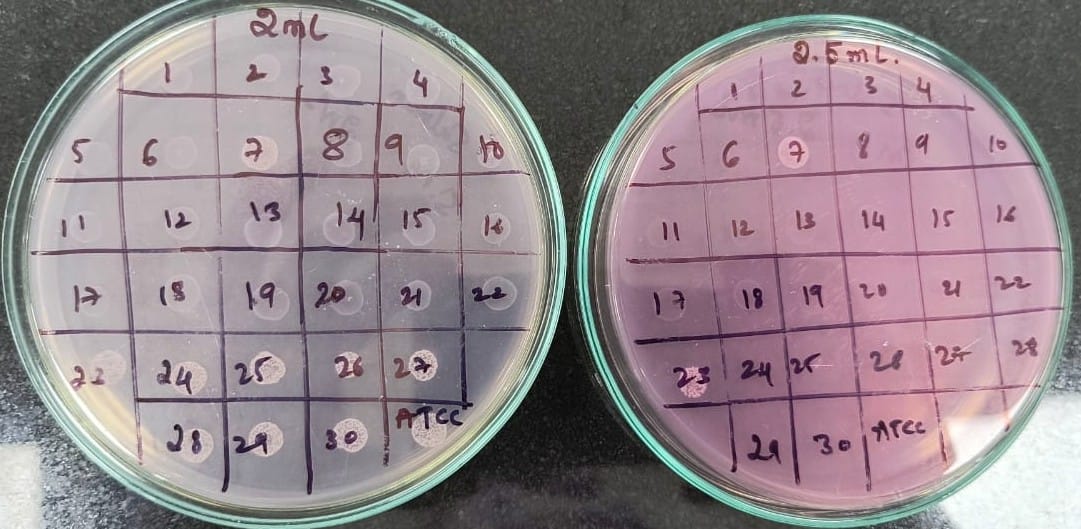

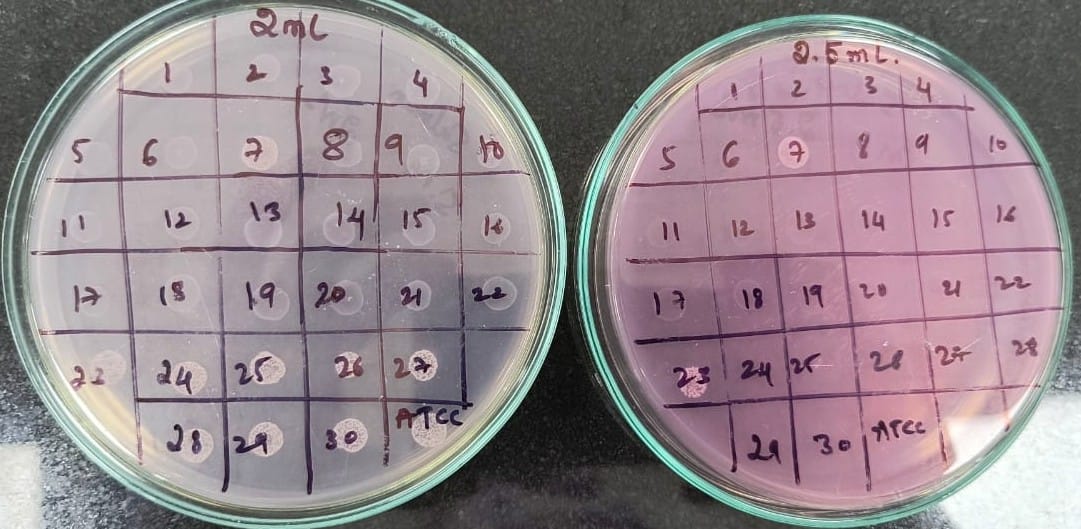

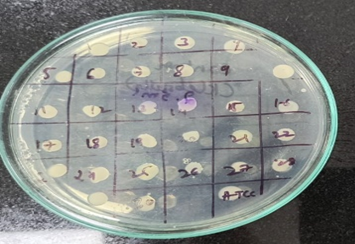


**Control 2mg/ml 2.5 mg/ml**

**B**. Increase in prodigiosin concentration inhibits the growth of *E. faecalis* isolates and Reference strain

**Figure S3**: A representative picture showing effect of different concentration of pigments on *Candida* spp. as determined by agar dilution method


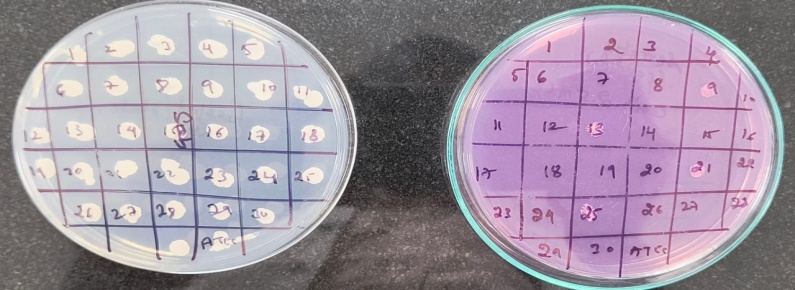

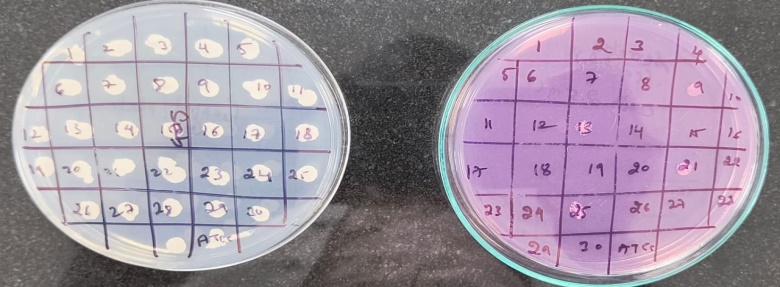

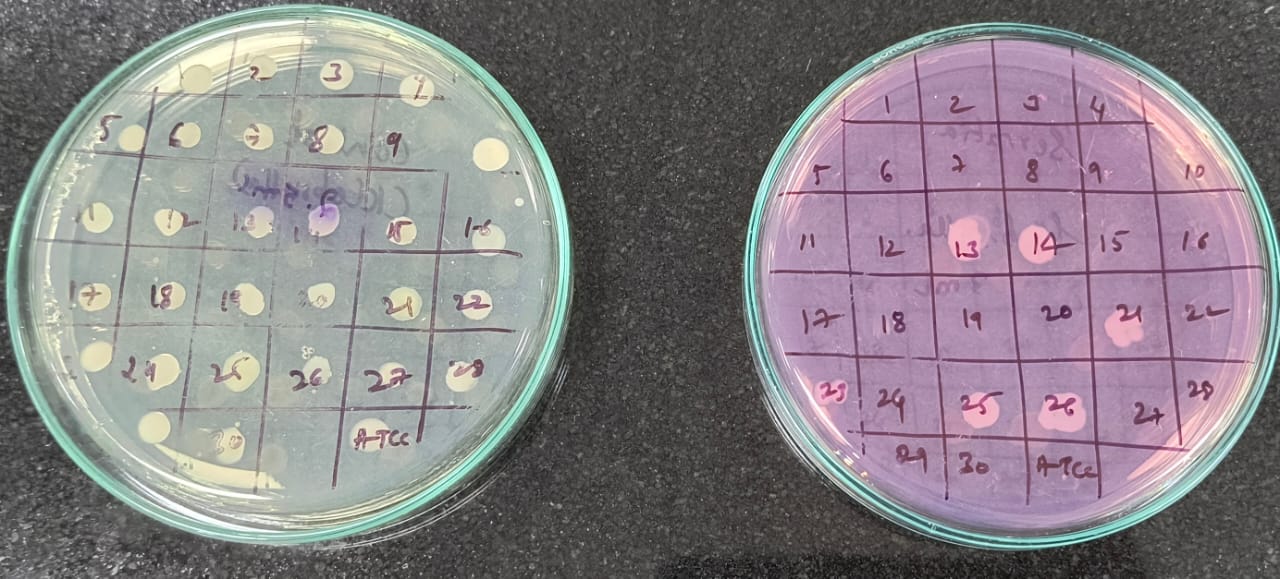


**B.** Increase in prodigiosin concentration inhibits the growth of *Candida* spp*.* and Reference strain

**Control 2.5mg/ml 4mg/ml**


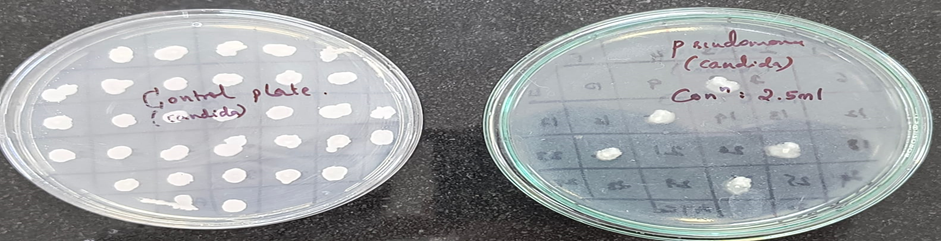

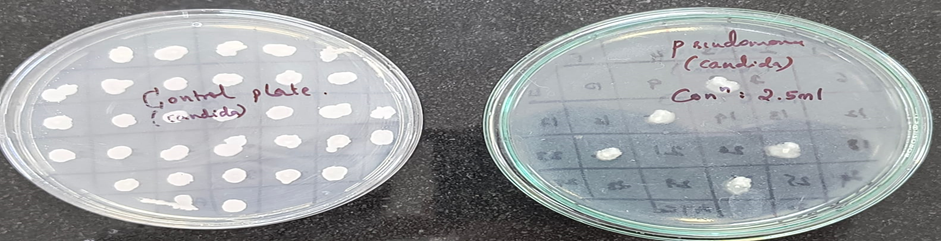

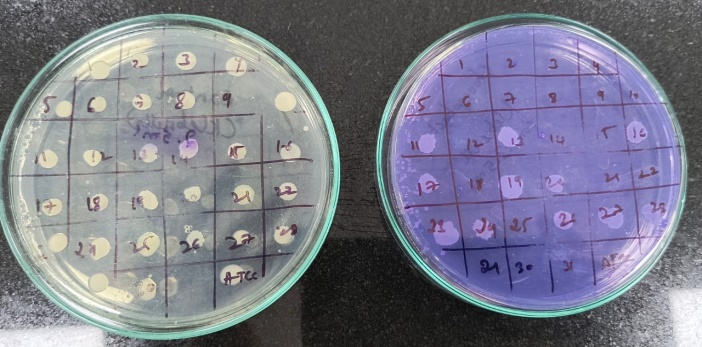


**Control 2mg/ml 7mg/ml**

**A**. Increase in pyocyanin concentration inhibits the growth of *Candida* spp*.*and Reference strain


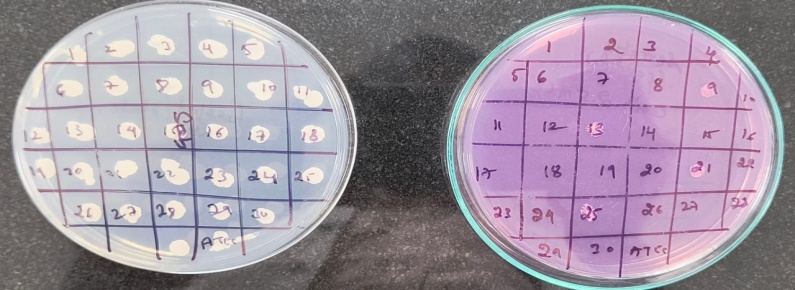

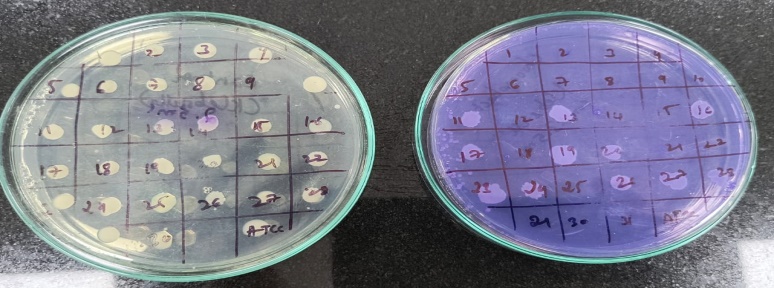


**Control 3mg/ml 5mg/ml**

**C**. Increase in violacein concentration inhibits the growth of *Candida* spp*.* isolates and Reference strain


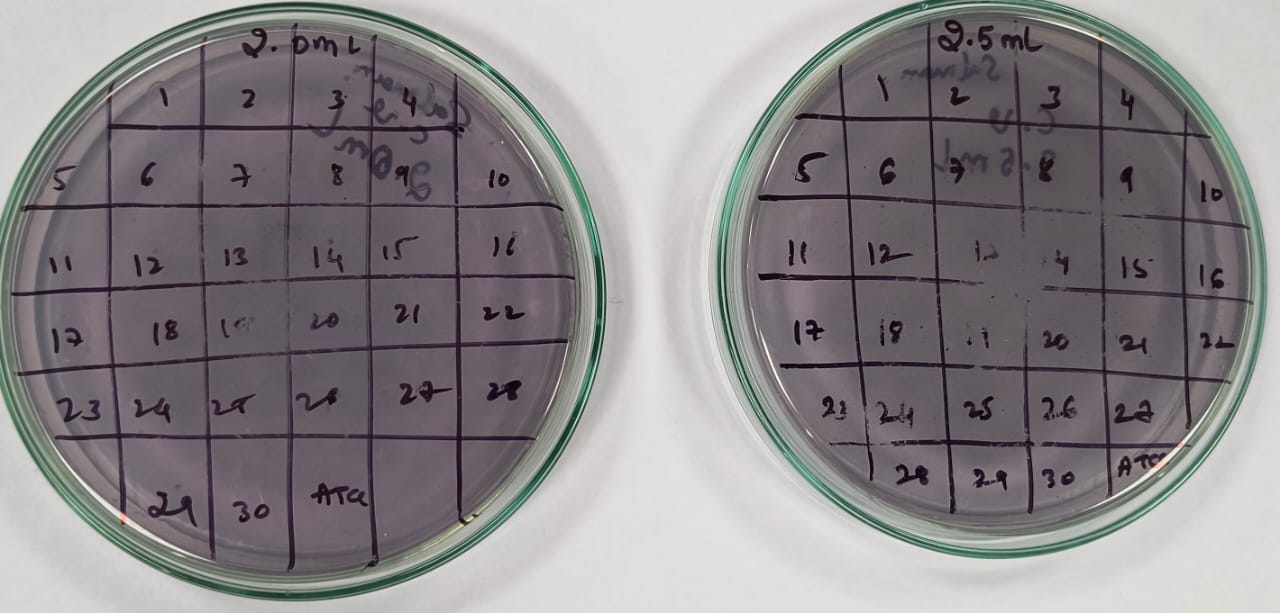

Supplement: Supporting Information — Additional supporting information can be found online in the Supporting Information section. [file 3951925.f1.docx]
